# Bilateral Choanal Atresia in a Newborn With Trisomy of Chromosome 21: A Case Report

**DOI:** 10.7759/cureus.115415

**Published:** 2026-08-29

**Authors:** Thiago H Gouveia, Vanessa D Rodrigues, Maria Vitória C Sousa, Andrea O Cecchi, Renato T Barufi

**Affiliations:** 1 Department of Medicine, University of Franca, Franca, BRA; 2 Department of Medicine, Atenas University, Paracatu, BRA

**Keywords:** choanal atresia, choanal narrowing, down syndrome, otorhinolaryngological diseases, pediatric airway obstruction

## Abstract

The aim of this study is to report the case of a newborn with bilateral choanal atresia (CA) associated with trisomy of chromosome 21, highlighting the clinical, diagnostic, therapeutic, and prognostic aspects of this rare condition, in order to contribute to the knowledge and management of similar cases. The case involved a late preterm female newborn, White, born by vaginal delivery in a city in the interior of the state of São Paulo, weighing 3,120 g, with a head circumference of 33 cm. At birth, she presented cyanosis, absence of cry, hypotonia, and bradycardia, requiring ventilation and orotracheal intubation. The unsuccessful attempt to pass a nasogastric tube suggested nasopharyngeal obstruction. Since birth, she presented phenotypic features typical of Down syndrome, including low-set ears, dorsal gibbus, and epicanthal folds. Imaging examinations confirmed bilateral CA, and karyotyping confirmed trisomy of chromosome 21. She underwent surgical correction, with satisfactory initial progress, and was discharged after 28 days. Two months later, she presented with recurrence of choanal synechia and underwent reoperation for airway recanalisation. Therefore, the association between bilateral CA and Down syndrome is rare and carries a significant neonatal respiratory impact. Early diagnosis and a multidisciplinary approach are essential to reduce complications and improve prognosis.

## Introduction

Choanal atresia (CA) is a complete blockage between the posterior nasal cavity and the nasopharynx that can cause severe respiratory obstruction symptoms in newborns. Obstructive symptoms occur shortly after birth, since neonates are considered obligate nasal breathers due to the anatomical relationship between the oral cavity, oropharynx, and tongue. Immediately following umbilical cord clamping, neonates affected by bilateral CA may develop severe respiratory distress [1].

Unlike orofacial clefts, defects in the nasal cavity and airway formation, such as CA and choanal stenosis (CS), have been insufficiently studied. CA, characterized by congenital obstruction of the posterior nasal aperture, preventing communication between the nasal cavity and the nasopharynx, occurs in approximately 1 in 5,000-8,000 live births [1-2]. Although it may occur as an isolated anomaly, CA is frequently associated with congenital syndromes, particularly CHARGE syndrome and other craniofacial or chromosomal disorders [1-2]. Both CA and CS require immediate intervention and have a significant impact on the quality of life of affected individuals [3]. 

CA may occur either as an isolated malformation or in association with other congenital anomalies. CHARGE syndrome (coloboma, heart disease, atresia of the choanae, retarded growth and mental development, genital anomalies, and ear malformations and hearing loss) is the most frequently associated syndrome, although associations with Treacher Collins syndrome (mandibulofacial dysostosis) and other craniofacial or chromosomal disorders have also been described [1-2]. The association with trisomy 21 is rarely reported in the major scientific literature databases, which reinforces the relevance of publications on this topic. This association is clinically relevant because individuals with Down syndrome frequently present craniofacial and upper airway characteristics, including a flattened nasal bridge, midface and mandibular hypoplasia, relative macroglossia, and narrowing of the nasopharyngeal region, which may predispose to upper airway obstruction and complicate respiratory management [4-5]. 

With the aim of improving the patient's condition, surgery is recommended. In cases of unilateral atresia, there is a consensus in the literature to wait between six and 24 months, which may be postponed in cases of worsening respiratory symptoms. For patients with bilateral atresia, surgery may be performed on a semi-elective basis following prior cardiological and anaesthetic evaluation, or in some cases immediately after diagnosis [1].

Given that CA can significantly compromise patient survival and impair child development, the publication of case reports becomes even more relevant, especially because many questions remain unanswered, such as the optimal timing for corrective surgery. These reports therefore aim to explore and discuss diagnostic methodologies, treatment options, and, above all, patient prognoses. In the present study, the coexistence of bilateral CA with Down syndrome makes the case particularly noteworthy, not because of the surgical technique itself, but because of the combination of an uncommon chromosomal condition with bilateral choanal obstruction and a complex postoperative course. The patient presented with mixed bony and membranous CA, early postoperative restenosis requiring surgical reintervention, and subsequent difficulty in maintaining a stable airway that ultimately required tracheostomy. These features illustrate how the management of bilateral CA may become particularly challenging in a newborn with trisomy 21 and associated craniofacial and airway abnormalities. Thus, the educational value of this case lies in highlighting the interaction between the underlying chromosomal condition, upper-airway anatomy, postoperative restenosis, and the need for prolonged and multidisciplinary airway management, contributing to the clinical understanding of this uncommon association.

## Case presentation

A female newborn, daughter of a 31-year-old mother (G2P2A0), with a gestational age of 41 weeks, born by uncomplicated vaginal delivery in a city in the interior of the state of São Paulo. Birth weight was 3,120 g, length 47 cm, head circumference 33 cm, and chest circumference 32.5 cm. Apgar scores were 4 at one minute, 8 at five minutes, and 8 at 10 minutes [6]. Shortly after birth, she developed absence of cry, generalized hypotonia, and heart rate below 100 bpm, requiring positive pressure ventilation for four minutes without satisfactory response, which prompted orotracheal intubation in the delivery room.

Following intubation, cardiopulmonary status improved. An extubation attempt was made but was unsuccessful due to immediate desaturation, necessitating reintubation. Given the persistent need for ventilatory support, the patient was transferred to the neonatal intensive care unit for continuous monitoring, with an initial diagnosis of a term newborn, appropriate for gestational age, with early respiratory distress and craniofacial features suggestive of a genetic syndrome, including low-set ears and relative macroglossia [7]. 

On physical examination at admission, she was receiving mechanical ventilation, with oxygen saturation of 92% on a fraction of inspired oxygen of 28%, mean heart rate of 140 bpm, axillary temperature of 36.5°C, and blood pressure within expected parameters for age. She presented phenotypic features suggestive of Down syndrome, including a flat nasal bridge, relative macroglossia, low-set ears, and a single palmar crease. Nasal inspection revealed no perceptible airflow, even after careful aspiration. Pulmonary auscultation demonstrated present and well-distributed breath sounds without adventitious sounds, while cardiac auscultation showed regular rhythm, normal heart sounds, and no murmurs. The abdomen was soft, non-tender to palpation, with no visceromegaly or palpable masses.

The association between the absence of nasal airflow and episodes of cyanosis that improved with crying and worsened with closure of the oral cavity during ventilation attempts raised the hypothesis of a congenital malformation of the upper airways. On the second day of life, assessment by the Otorhinolaryngology team was requested, which indicated bedside nasofibroscopy. The examination demonstrated adequate initial progression of the endoscope through the nasal fossae; however, complete obstruction was found at the transition to the nasopharynx, associated with the presence of thick secretions and rigid-consistency tissue, preventing further advancement of the instrument and complete assessment of the nasal anatomy (Figures 1, 2).

**Figure 1:**
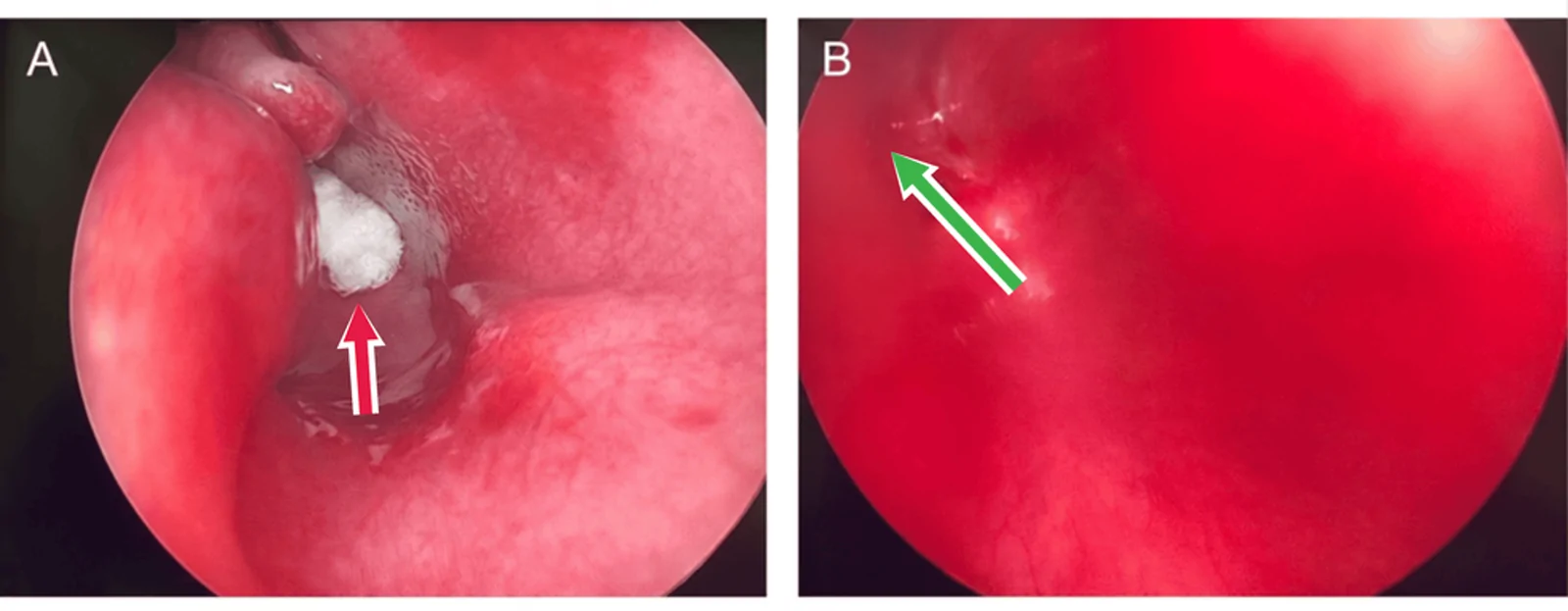
Right nasal cavity (A) with confirmed choanal atresia. Clear view of the atretic plate in the right nasal cavity (B). Red (A): atretic plate. Green (B): atretic plate (detail).

**Figure 2:**
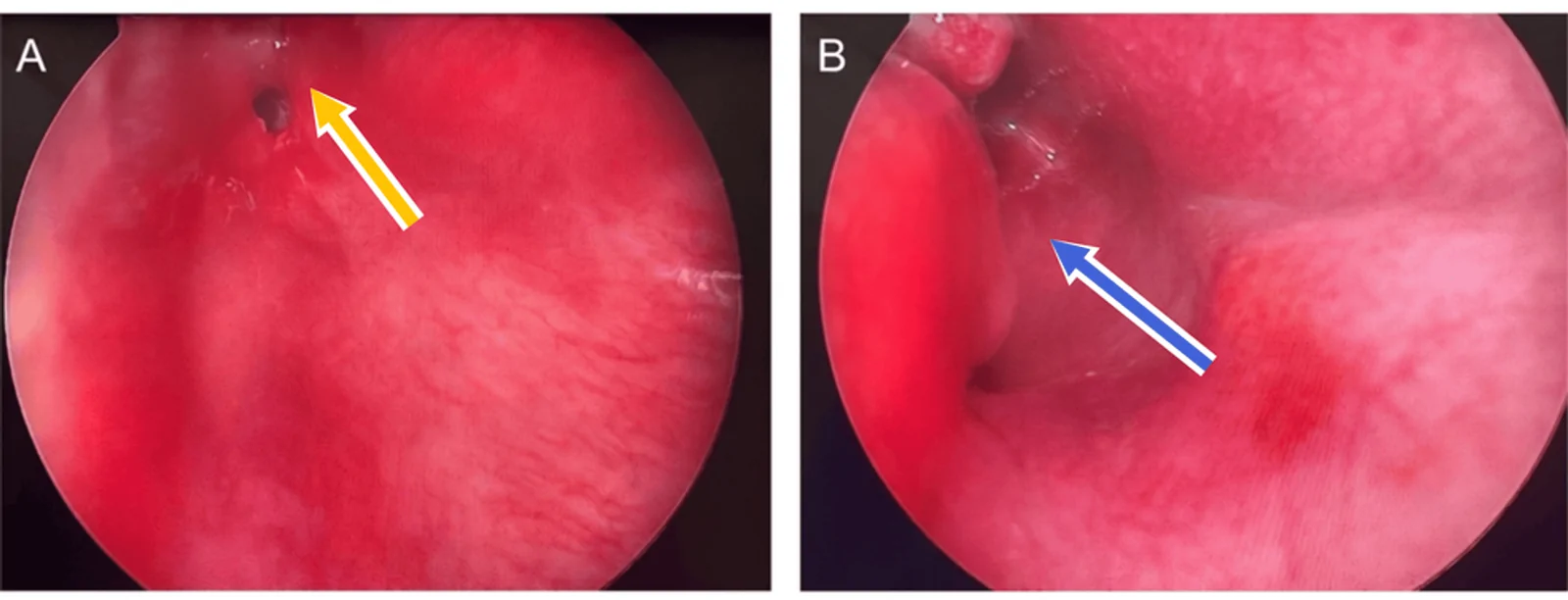
Endoscopic image of the left nasal cavity showing absence of visualisation of the posterior choanal lumen, a finding suggestive of choanal atresia. Endoscopic image of the right nasal cavity showing absence of the posterior choanal lumen, a finding suggestive of choanal atresia. Yellow (A): absence of the choanal lumen (left). Blue (B): absence of the choanal lumen (right).

Given the diagnostic limitation, computed tomography (CT) of the skull and face was performed, revealing bony narrowing of the right nasal choana secondary to vomer thickening, with hypoattenuating content in its interior, compatible with bony choanal atresia. On the left side, obstruction by hypoattenuating material was observed, suggesting a membranous component. The nasal septum was midline, and the ethmoid fossae were symmetrical. The combination of findings confirmed the diagnosis of bilateral choanal atresia, bony on the right and predominantly membranous on the left (Figure 3).

**Figure 3:**
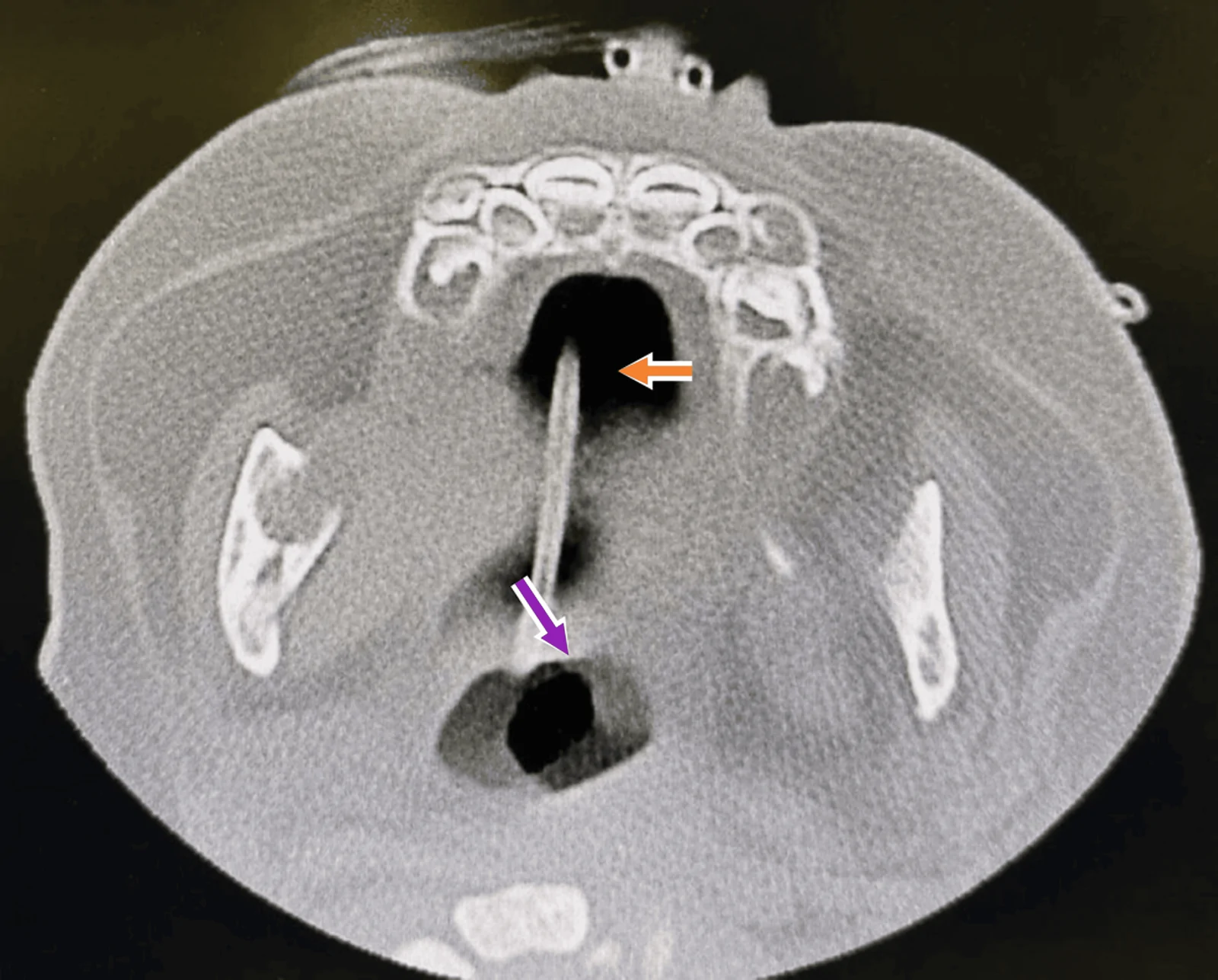
2D axial section that allows visualization of the internal airway and clearly shows (including the marking) the bony narrowing and thickening of the vomer. Orange: thickened vomer. Purple: bony narrowing of the right choana.

Given the ventilatory dependence, early surgical intervention was indicated. On the seventh day of life, the patient underwent transnasal endoscopic correction, with removal of the atretic plate, opening of the choanae, and dilatation of the posterior airway (Figure 4).

**Figure 4:**
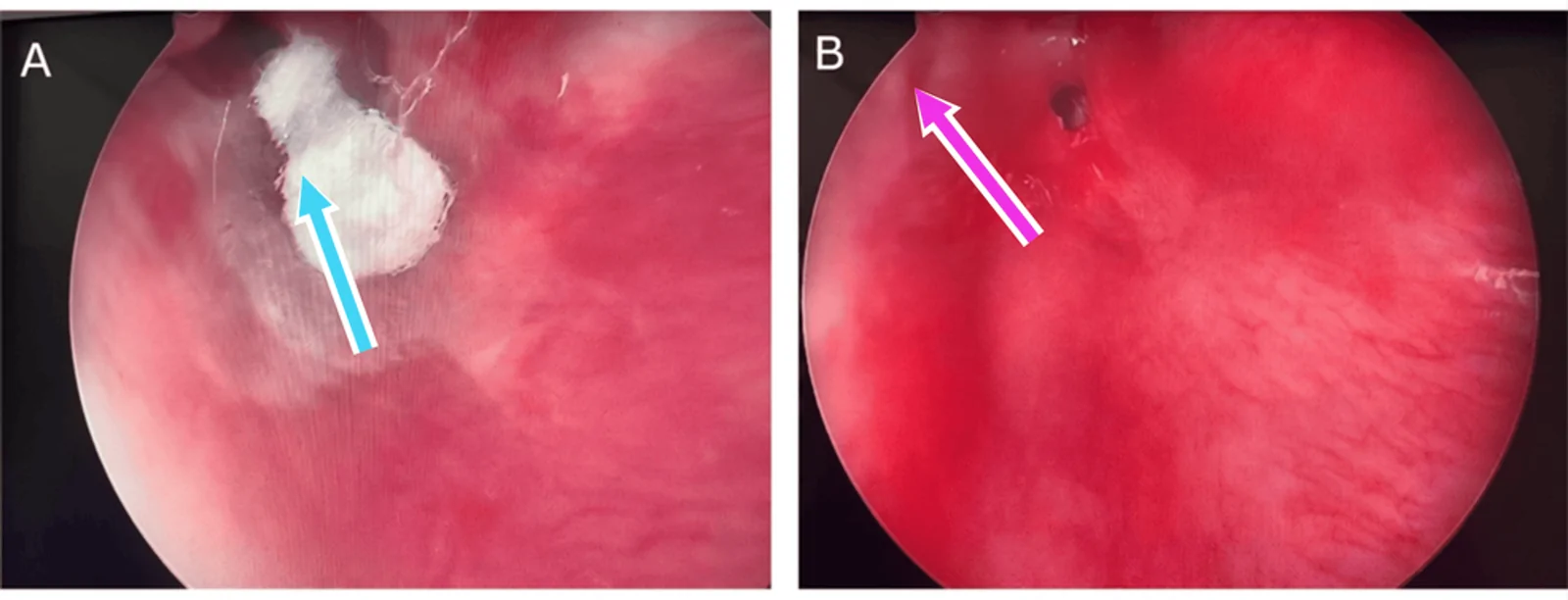
Left nasal cavity (A) with confirmed choanal atresia. Post-dissection of the left nasal cavity (B) - creation of an opening. Cyan (A): atretic plate. Magenta (B): opening created.

At the end of the procedure, a bilateral communicating stent was positioned with the aim of maintaining airway patency and reducing the risk of cicatricial restenosis (Figure 5).

**Figure 5:**
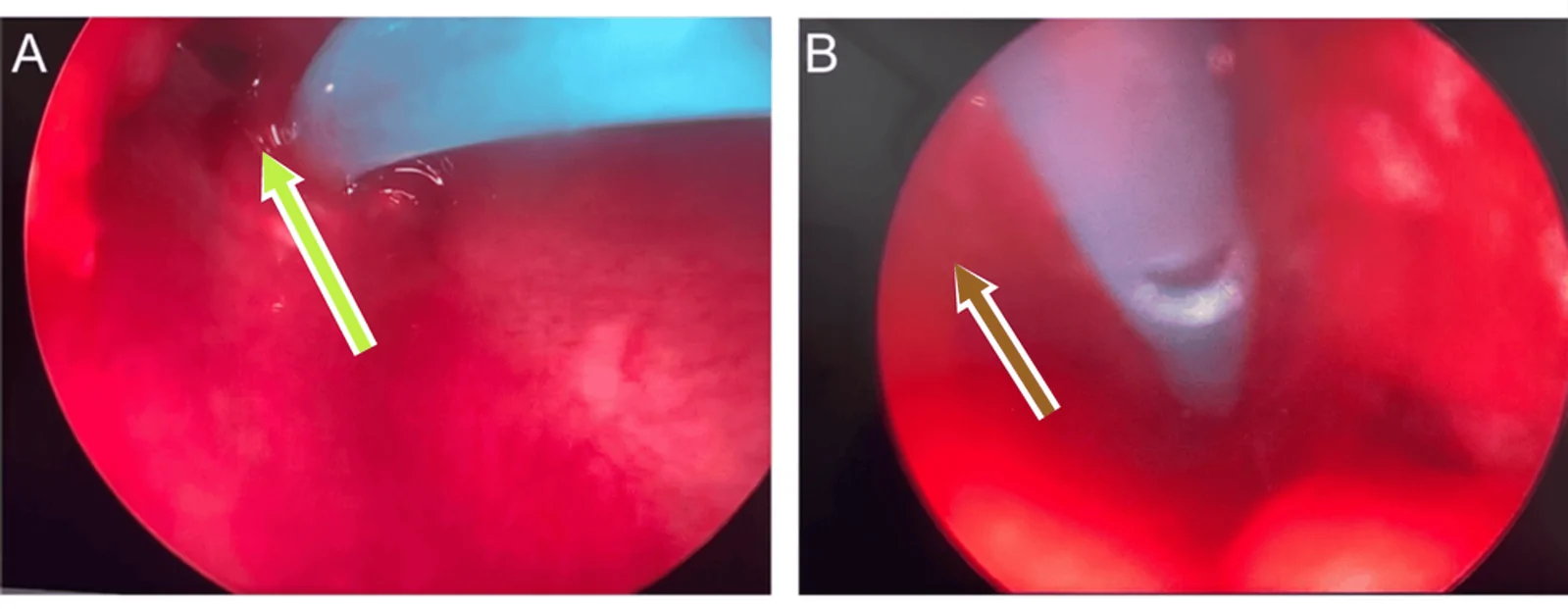
Verification of patency of the left nasal cavity (A) with a catheter. Verification of patency of the right nasal cavity (B) with a catheter. Lime green (A): catheter (patency, left nasal cavity). Brown (B): catheter (patency, right nasal cavity).

However, in the subsequent days, progressive difficulty in managing the stent was observed, along with sporadic episodes of nasal bleeding during aspiration. On the 12th day of life, the stent could no longer be advanced through the nasal cavities, associated with a new episode of respiratory distress. A new nasofibroscopy revealed minimal choanal opening on the left and absence of the choanal lumen on the right, characterizing early recurrence due to restenosis.

The patient began requiring frequent aspiration due to secretion accumulation and progressive upper airway obstruction. On the 23rd day of life, a new endoscopic examination revealed the presence of hematic crusts, clots, and mucosal irregularity of the right nasal cavity, hindering instrument progression and necessitating renewed orotracheal intubation. Given the difficulty of prolonged airway maintenance and the risk of subglottic stenosis associated with intubation, a tracheostomy was performed using an appropriately sized pediatric cannula, a procedure conducted without complications and with subsequent improvement in respiratory stability.

New CT of the paranasal sinuses demonstrated persistent significant narrowing of the choanae, with partial obliteration of the nasal cavities, reinforcing the diagnosis of postoperative stenosis and indicating the need for a new surgical intervention (Figure 6). The second surgical approach was performed at 11 weeks of life using the same endoscopic technique, without complications, with immediate extubation and maintenance of adequate oxygen saturation.

**Figure 6:**
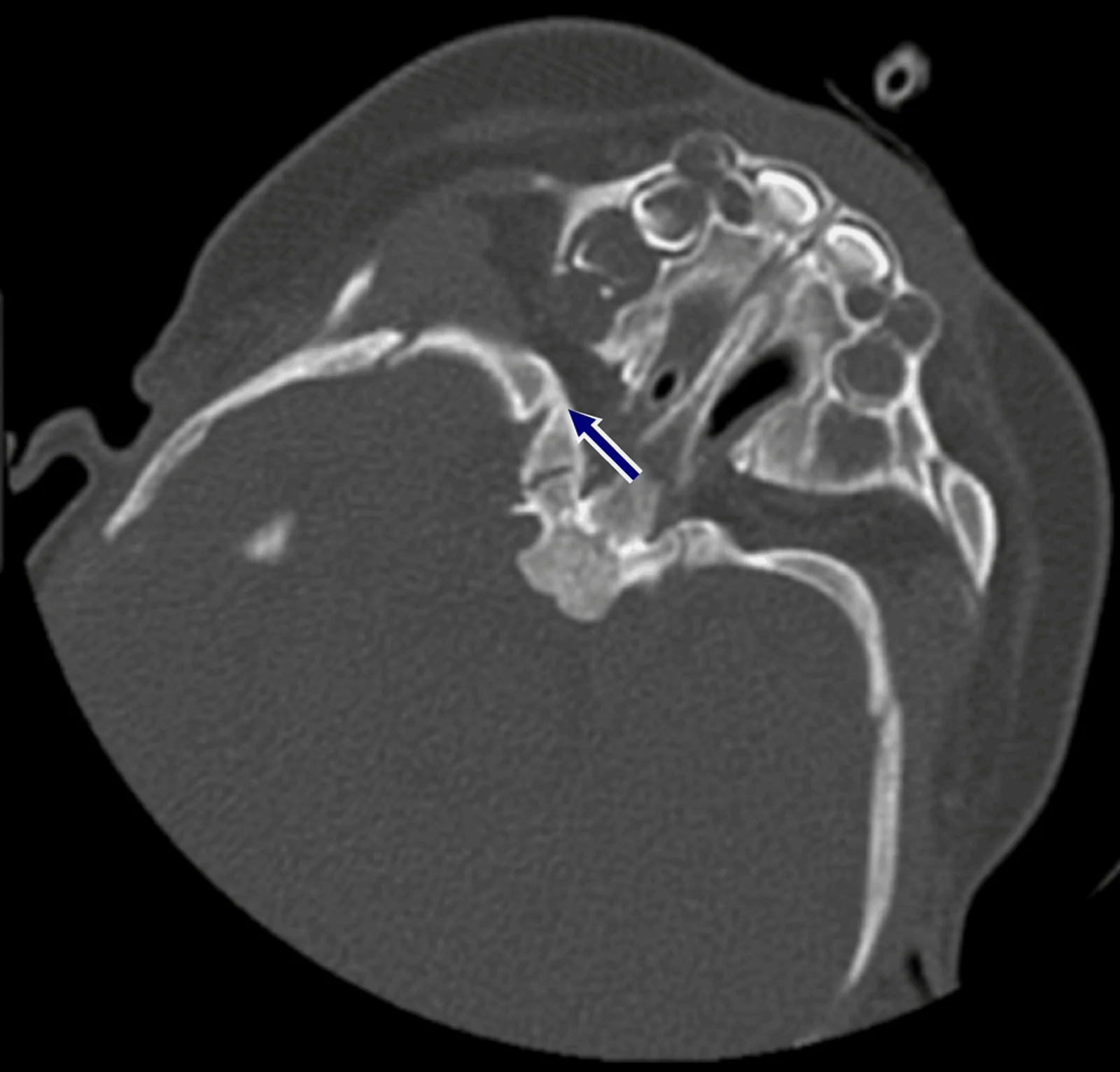
CT of the paranasal sinuses demonstrating persistent significant narrowing of the choanae with partial obliteration of the nasal cavities. Navy blue: persistent narrowing of the choanae

In the postoperative period, the patient remained under intensive follow-up with continuous monitoring and regular respiratory physiotherapy. She showed progressive improvement in the ventilatory pattern, although she still presented occasional desaturation episodes associated with thick secretion accumulation. Saline solution instillation and monitored aspiration were implemented as adjunctive measures to maintain airway patency and optimize the healing process (Figure 7).

**Figure 7:**
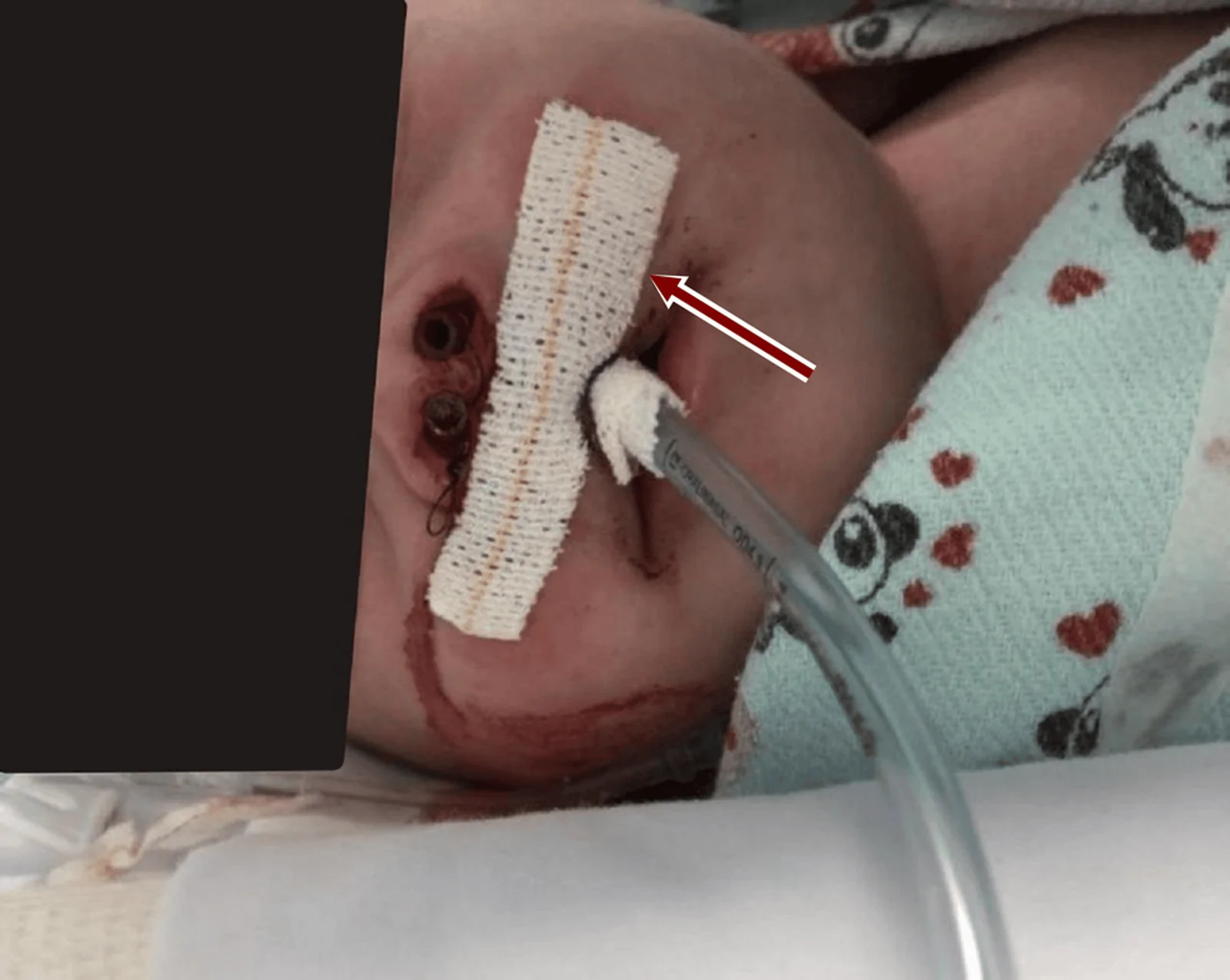
Postoperative clinical appearance showing the nasal device in position, used to maintain airway patency. Burgundy: nasal device (stent)

Following clinical stabilisation, she was transferred from the intensive care unit to the ward, where she remained for approximately two weeks under observation, with minimal respiratory support and multiprofessional follow-up. During this period, she showed favorable progress, with progressive improvement in the respiratory pattern and adequate adaptation to the reconstructed airway, without hemodynamic instability.

Given the satisfactory clinical course, she was discharged with specific instructions for home care, including tracheostomy management, nasal hygiene, and maintenance of airway patency. Outpatient follow-up was maintained with Otorhinolaryngology, Pediatrics, and Physiotherapy teams, initially at short intervals and subsequently at longer intervals as clinical stability was achieved. The patient showed good adherence to follow-up and satisfactory respiratory progress throughout.

## Discussion

Bilateral CA, as observed in the described case, can be classified as mixed, given the presence of both bony and membranous components. This condition represents a neonatal emergency, since newborns are predominantly obligate nasal breathers in the first months of life. Thus, nasal obstruction can compromise not only breathing but also, in the short and long term, sucking, growth, and craniofacial development [1-9]. The association with genetic syndromes is described in the literature, with CHARGE syndrome being the most frequently reported [1-2]. Although rare, the association with other chromosomal conditions has been described in case reports, both in the bilateral and unilateral forms of CA in patients with Down syndrome [10-11]. In addition, in a recent retrospective study, 13 of 84 patients who underwent surgical correction of choanal narrowing, corresponding to 15.5%, had Down syndrome [12].

In the present case, the newborn exhibited severe symptoms, including immediate respiratory distress at birth, absence of nasal airflow, and persistent ventilatory dependence, a clinical presentation typical of bilateral CA, which facilitated early diagnosis. However, in situations where the choanal obstruction is partial, intermittent, or unilateral, symptoms tend to be milder and non-specific, potentially delaying diagnosis to later stages of development. These findings, particularly the cyanosis, which showed intermittent improvement with crying, when oral breathing occurred, and worsening with closure of the oral cavity, further reinforced the diagnostic suspicion of significant nasal obstruction [9]. Therefore, the clinical presentation described in this report primarily represents severe bilateral choanal obstruction in the neonatal period and should not be extrapolated to unilateral or partial forms, which may present with milder or delayed symptoms.

From a diagnostic standpoint, nasofibroscopy combined with CT of the skull and face enabled precise identification of bilateral CA, with a bony component on the right and a membranous component on the left, an anatomical pattern that predominates in up to 70% of cases [1-13]. Tomographic confirmation was essential for surgical planning and definition of the therapeutic approach. Previous reports have described surgical correction of bilateral CA between the 10th and 13th day of life, including in premature infants [14-16]. In the present case, surgery was performed on the seventh day of life because of the severity of the respiratory condition and persistent ventilatory dependence. The timing of surgery was individualized according to the patient's clinical condition. Furthermore, it is important to highlight that patients with Down syndrome frequently present structural anatomical alterations of the airways, such as nasal narrowing, a flat nasal bridge, midface hypoplasia, macroglossia, and adenotonsillar crowding, which may contribute to respiratory compromise and favour upper airway obstruction [4]. In this context, a retrospective study conducted at a university hospital in Belgium demonstrated that airway abnormalities were identified in 71% of children with Down syndrome who underwent bronchoscopy for chronic respiratory symptoms, compared with 32% in a control group of children with similar symptoms but without the syndrome [5].

The surgical technique employed was transnasal endoscopic repair, which is strongly recommended in cases of bilateral CA [2]. This technique allows direct visualisation of the atretic area and elevation of mucosal flaps to cover the raw surfaces, with the aim of reducing the occurrence of restenosis. It is also considered less invasive and associated with lower complication rates when compared with the transpalatal approach, which, despite providing greater surgical exposure, tends to be more aggressive as it involves perforation of the hard palate and carries a higher risk of postoperative complications [9]. Among the surgical alternatives, techniques that avoid postoperative stenting include endoscopic repair with posterior vomer resection [17]. Although stentless repair may be associated with fewer complications, particularly granulation tissue formation, current evidence has not demonstrated a statistically significant difference in choanal patency between stented and stentless approaches [18]. However, even with an appropriate approach, the clinical course revealed early restenosis, one of the most commonly described complications in the literature, particularly in bilateral cases and in neonates with complex anatomical abnormalities [19].

The need for surgical reintervention and subsequent tracheostomy in the present case reflects the difficulty in maintaining airway patency in neonates with bilateral CA. Furthermore, the literature demonstrates that patients with this form of the disease frequently require multiple surgical procedures throughout treatment, regardless of the technique employed, reinforcing the complexity of management and the importance of long-term follow-up [8]. Postoperative management of bilateral CA will depend primarily on associated conditions, the gestational age of the newborn, and the treatment of other comorbidities. Premature infants and/or those with CHARGE syndrome and intubated patients tend to have a higher risk of postoperative complications, and consequently longer hospital stays [15-20]. Moreover, monitoring of respiratory patterns after surgery until hospital discharge is necessary, with the aim of ensuring not only adequate breathing but also nutrition. In addition, parents must be instructed regarding surgical site hygiene and how to respond in cases of respiratory distress [16]. It is also worth noting that CT or other radiological examinations are not routinely indicated in postoperative follow-up, even in the presence of restenosis [2].

## Conclusions

This case report highlights the relevance of the association between bilateral CA and Down syndrome, an infrequent but described relationship in the literature that should be considered in the presence of suggestive phenotypic findings. Early identification of this association enables not only adequate airway management but also the implementation of a multidisciplinary approach from the neonatal period. As a single case report, these findings cannot establish the optimal timing of surgical correction or predict long-term outcomes. Despite this limitation, the detailed description contributes to expanding knowledge of this specific clinical presentation and reinforces the need for vigilance regarding the recurrence of nasal obstruction.
